# Chemical Composition and *in-Vitro* Evaluation of the Antimicrobial and Antioxidant Activities of Essential Oils Extracted from Seven *Eucalyptus* Species

**DOI:** 10.3390/molecules201119706

**Published:** 2015-11-18

**Authors:** Abdul Ghaffar, Muhammad Yameen, Shumaila Kiran, Shagufta Kamal, Fatima Jalal, Bushra Munir, Sadaf Saleem, Naila Rafiq, Aftab Ahmad, Iram Saba, Abdul Jabbar

**Affiliations:** 1 Department of Applied Chemistry and Biochemistry, Government College University, Faisalabad 38000, Pakistan; yaminsynergic@gmail.com (M.Y.); shumaila.asimch@gmail.com (S.K.); shagufta_kamal@yahoo.com (S.K.); bushramunirje@hotmail.com (B.M.); 2 Department of Zoology and Fisheries, Government College University, Faisalabad 38000, Pakistan; Fatima_jalal@hotmail.com; 3 Department of Chemistry, Government College for Women University, Faisalabad 38000, Pakistan; sadafsaleemuaf@gmail.com (S.S.); n_rafiq2005@hotmail.com (N.R.); 4 Department of Biochemistry/US-Pakistan Center for Advanced Studies in Agriculture and Food Security (USPCAS/AFS), University of Agriculture, Faisalabad 38000, Pakistan; ahmadaftab1862@hotmail.com; 5 Department of Chemistry, Government College University, Faisalabad 38000, Pakistan; iramsaba2005@gmail.com

**Keywords:** *Eucalyptus* essential oils, GC/MS analysis, antimicrobial, antioxidant

## Abstract

*Eucalyptus* is well reputed for its use as medicinal plant around the globe. The present study was planned to evaluate chemical composition, antimicrobial and antioxidant activity of the essential oils (EOs) extracted from seven *Eucalyptus* species frequently found in South East Asia (Pakistan). EOs from *Eucalyptus citriodora, Eucalyptus melanophloia*, *Eucalyptus crebra*, *Eucalyptus tereticornis*, *Eucalyptus globulus*, *Eucalyptus camaldulensis* and *Eucalyptus microtheca* were extracted from leaves through hydrodistillation. The chemical composition of the EOs was determined through GC-MS-FID analysis. The study revealed presence of 31 compounds in *E. citriodora* and *E. melanophloia*, 27 compounds in *E. crebra*, 24 compounds in *E. tereticornis*, 10 compounds in *E. globulus*, 13 compounds in *E. camaldulensis* and 12 compounds in *E. microtheca*. 1,8-Cineole (56.5%), α-pinene (31.4%), citrinyl acetate (13.3%), eugenol (11.8%) and terpenene-4-ol (10.2%) were the highest principal components in these EOs. *E. citriodora* exhibited the highest antimicrobial activity against the five microbial species tested (*Staphylococcus aureus*, *Bacillus subtilis*, *Escherichia coli*, *Aspergillus niger* and *Rhizopus solani*). Gram positive bacteria were found more sensitive than Gram negative bacteria to all EOs. The diphenyl-1-picrylhydazyl (DPPH) radical scavenging activity and percentage inhibition of linoleic acid oxidation were highest in *E. citriodora* (82.1% and 83.8%, respectively) followed by *E. camaldulensis* (81.9% and 83.3%, respectively). The great variation in chemical composition of EOs from *Eucalyptus*, highlight its potential for medicinal and nutraceutical applications.

## 1. Introduction

The interest in use of natural medicines from plants has been increasing in the past few years in industrialized societies, particularly against microbial agents because of the ever growing problem of antibiotic resistance [1]. The applications of essential, volatile and hydrophobic oils from natural resources are rapidly increasing in medicinal, food and cosmetic industries to replace harmful synthetic additives [2,3]. The EOs are aromatic essences usually derived from the aerial parts of the plants containing oxygenated compounds; phenols, alcohols, esters, ethers, ketones, aldehydes and oxides, hydrocarbons; terpenes, organic sulphur and nitrogenous compounds and benzene derivatives [4,5]. EOs used as food flavorings and preservatives are generally recommended as safe (GRAS) and have broad spectrum of activity against different bacterial and fungal strains due to the presence of high percentages of monoterpenes, eugenol, cinnamic aldehydes, thymol and polyphenols [6,7]. 

*Eucalyptus*, with almost 900 species, is found worldwide, with some species being native to South East Asia. Due to its rapid growth *Eucalyptus* is an important hardwood forest crop used in the pulp and paper industry. Its timber is used for construction and fuel [8], while the gum is used for the treatment of diarrhoea and as an astringent in dentistry [9]. More than 300 species of this genus contain volatile oils in their leaves [10]. Worldwide production of *Eucalyptus* EOs is 3000 tons and major producers of EOs of *Eucalyptus* are China, Spain, Portugal, South Africa and Chile [11]). *Eucalyptus* EOs are used as fragrance elements in household products and cosmetics such as soaps, detergents, lotions and perfumes. They are also used as flavor elements in foods and beverages such as baked goods, confectionaries, meat products, ice creams and soft drinks. In addition their use to help prevent and treat human diseases has undergone a relatively recent revival and expansion [12].

*Eucalyptus* EO is officially listed in the Indian Pharmacopeia as a counterirritant and mild expectorant and also in the Chinese Pharmacopeia as a skin irritant. EOs of *Eucalyptus* have been extensively used as expectorant in cough and cold compounds in various oral dosage forms, including lozenges and syrups, and as an inhalant in vapour baths. It is also used externally for percutaneus absorption in dosage forms including the EOs, liniments and ointments [13]. EO of *Eucalyptus* is ingested orally to treat catarrh, used as inhalant and applied tropically as a rubefacient [14].

Volatile oils of *Eucalyptus* are used as antioxidants and anti-inflammatory agents for skin diseases, rheumatism and as expectorant in cases of bronchitis [15,16]. Many researchers have reported the chemical composition, antioxidant and antimicrobial activities of *Eucalyptus* species [17]. However, geographical distribution and species variation greatly affect these properties which require extensive studies to explore the potential of this plant. The chemical composition of eight *Eucalyptus* species’ essential oils from Tunisia reported the presence of 1,8-cineole as the major ingredient, followed by cryptone, α-pinene, *p*-cymene, α-terpineol, phellandral, cuminal, globulol, limonene, aromadendrene, sapthulenol and terpinene-4-ol [18]. 1,8-Cineole and α-pinene have been reported as major components of EOs from seven *Eucalyptus* species [19].

*Eucalyptus* was introduced in Pakistan long ago, but interest in the tree has increased considerably in the last two decades and lots of renewed efforts towards its propagation have been made. The large scale plantation of *Eucalyptus* in Pakistan has been recommended [20]. The growth of *Eucalyptus* in Pakistan is popular with public sector institutions, roadside plantations are widely distributed and it is being introduced to farmers in irrigated, saline and waterlogged soil areas as a purported cash crop. Its wood has been widely used for houses and furniture. However, its leaves are not yet used commercially. Currently Pakistan is importing more than 10 tons of *Eucalyptus* EOs from different countries as compared to 8.9 and 8.16 tons of *Eucalyptus* EOs in 1999–2000 and 2000–2001.

Presently, the volatile constituents and antioxidant activity of EOs from Pakistani *Eucalyptus* have not been rationally investigated and no systematic information about its chemical composition is available. To address this gap in our knowledge, the present study reports the chemical composition, antimicrobial and antioxidant activity of the EOs from seven *Eucalyptus* species for their potential use in food and medicine. The objectives of the study were: (i) the extraction of EOs from leaves of *Eucalyptus* and quantification of its yield; (ii) the identification and quantification of chemical compounds in each oil and (iii) determination of antioxidant and antimicrobial activities against some selected strains of bacteria and fungi.

## 2. Results and Discussion

### 2.1. Percentage Yield of EOs by Steam Distillation

The average yield of EOs from the different species of *Eucalyptus* was 1.84% (*w*/*w*) after 7 h of distillation. *E. globulus* had highest oil content (1.91%), while *E. melanophloia* contained least amount (1.73%). These findings are similar to results reporting a 2.4% yield of oil from *E. gillii* cultivated in Iran and 2.3% in Tunisia [21,22]. The small differences may be due to different climatic, geographic and ecological parameters.

### 2.2. Physiochemical Analysis of Essential Oils

The physiochemical properties of seven different *Eucalyptus* EOs revealed that initially *Eucalyptus* EOs were colourless, but after about 2 weeks they showed yellow colours (Table 1). All the EOs were soluble in 2 mL of 80% ethanol, but insoluble in water. The specific gravity of the EOs extracted from the seven *Eucalyptus* species ranged from 0.84 to 0.94. The boiling point of *E. melanophloia* EO was the highest at 178 °C, followed by *E. citriodora* (177 °C) and the lowest was for *E. globulus* (161 °C).

**Table 1:** Physiochemical properties of *Eucalyptus* essential oils.

Physiochemical Property	*E. citriodora*	*E. camaldulensis*	*E. crebra*	*E. tereticornis*	*E. globules*	*E. melanophloia*	*E. microtheca*
Percentage yield	1.82	1.90	1.84	1.83	1.89	1.73	1.84
Color	Pale yellow	Slightly yellowish	Light yellow	Yellow to orange	Colorless to pale yellow	Yellow to brown	Yellow reddish,
Odor	Citronellal odor	1,8 Cineole odor	Camphor odor	Cineole-pinene odor	Herbal odor	Pinene odor	Cymene odor
Solubility	Insoluble in water, soluble in alcohol	Insoluble in water, soluble in alcohol	Insoluble in water, soluble in alcohol	Insoluble in water, soluble in alcohol	Insoluble in water, soluble in alcohol	Insoluble in water, soluble in alcohol	Insoluble in water, soluble in alcohol
Boiling point (°C)	177 °C	178 °C	165 °C	173 °C	161 °C	174 °C	166 °C
Specific gravity	0.85	0.92	0.90	0.84	0.89	0.94	0.86
Refractive index	1.49	1.45	1.42	1.44	1.38	1.41	1.47

### 2.3. Chemical Nature of Essential Oils

The chemical composition of the seven selected *Eucalyptus* EOs revealed great variation in the concentration and types of constituents (Table 2). Identification of the components of EOs was based on their GC retention times and mass spectra, which were compared to the published data and spectra of respective standards to those constituents. The GC-MS analysis thus revealed the presence of 31 components in *E. citriodora* and *E. melanophloia*, 27 components in *E. crebra*, 24 components in *E. tereticornis*, 14 components in *E. globulus*, 13 components in *E. camaldulensis* and 12 components in *E. microtheca*. The results are comparable to the 25 major compounds at an average concentration greater than 0.9% ± 0.2% reported earlier in eight *Eucalyptus* species EOs. *E. gillii* EO contained 34 compounds as reported by another study [23].

Previous studies on four species of *Eucalyptus* grown in Tunisia reported the chemical compositions of *E. salubris* (27 compounds; 99.2%), *E. salmonophloia* (31 compounds; 99.2%), *E. oleosa* (32 compounds; 97.6%), and *E. gracilis* (18 compounds; 97.7%) [23].

*E. citriodora* and *E. melanophloia* EOs consisted of the highest number (31) of chemical compounds. The major components in EO of *E. citriodora* were citronellal (22.3%), citronellol (20%), patchoulene (9.4%), germacrene-D (7.5%), α-terpinol (6.3%), eugenol (3.9%), α-pinene (3.6%), and β-citronellal (3.2%). Previous studies on *E. citriodora* EO showed total 17 components out of which citronellal (21.2%) citronellol (12.2%) and isopulegol (11.9%) were listed as the main components [9]. Analysis of *E. melanophloia* EO revealed 31 components including α-pinene (16.0%), β-phellandrene (14.3%), farnesol (10.0%) and verbenol (9.2%). These results show a very efficient production of such compounds as compared to monoterpenoid oils, with α-pinene (1.6%), 1,8-cineole (0.2%–60%) and *p*-cymene (0.2%–21%) as the major constituents [24]. The chemical composition of *E. camaldulensis* EO showed 13 components, with major components such as linalool (17.0%), 1,8-cineole (16.1%), *p*-cymene (12.2%), β-farnesol (11.1%), γ-terpinene (10.7%), phenythyl acetate (7.1%), geranial (6.6%), terpinene-4-ol (5.3%), paraldehyde (5.3%) and *p*-ment-1-en-3, 8-diol (5.1%). EO from *E. oleosa* stem, immature flowers and the fruit showed 1,8-cineole as principal compound, representing 31.5%, 47.0% and 29.1%, respectively [25]. The results show small variation from a previous study describing a total of 11 components out of which *p*-cymene (17.4%–28.6%), β-phellandrene (12.3%–14.4%) and β-pinene (0.9%–11.4%) were the main ingredients [26] and great variation from the 1,8-cineole (21.7%), β-pinene (20.5%) and methyl eugenol (6.1%) levels in *E. camaldulensis* [27]. The study is also comparable to a *E. camaldulensis* EO analysis reporting 1,8-cineole (45.7%) and *p*-cymene (17.1%) as major compounds obtained by hydrodistillation and 8,14-cedrane oxide (43.8%) and elemol (6.3%) by supercritical fluid extraction [28]. The findings of present study show a greater number of oxygenated compounds. The differences may be attributed to geographical, environmental and climatic variations affecting chemical composition of *Eucalyptus* species.

*E. crebra* oil was composed of 27 components. Limonene (14.3%) was the major constituent, followed by terpinene-4-ol (10.2%) and citrinyl acetate (9.1%). *E. tereticornis* EO showed 24 components with 1,8-cineole (15.2%), α-pinene (12.1%), myrtenal (8.1%), linalool, (7.4%) and paraldehyde nitrile (7.1%) as the most abundant. Another study reported that *E. tereticornis* EO contained 19 components out of which α-pinene (21.4%) was the major constituent but 1,8 cineole was absent [29]. In this study 1,8-cineole was the principal component (56.5%) found in *E. globulus* along with limonene (28.0%), α-pinene (4.2%), α-terpinol (4.0%) and globulol (2.4%). The present study shows higher contents of these compounds as compared to earlier reports for a total of 10 components in this plant, out of which 1,8 cineole (17.5%), α-pinene (1.7%) and α-phellandrene (1.1%) were the major constituents [30]. *E. microtheca* EO showed total 12 components, with α-pinene (31.4%), citrinyl acetate (13.2%), cymene (12.4%), eugenol (11.8%) and eucalyptol (8.1%) as the major ones. The results of the present study showed higher amounts than the cymene (10.3%) and α-pinene (10.0%) as the major constituents reported previously [31]. The chemical composition of EOs was statistically significantly different in all *Eucalyptus* species. Some components were found in abundance in some species and absent in some species.

**Table 2:** Relative percentage chemical composition of essential oils of the EOs extracted from seven *Eucalyptus* species.

Serial No.	Components	*E. citriodora*	*E. camaldulensis*	*E. crebra*	*E. tereticornis*	*E. globules*	*E. melanophloia*	*E. microtheca*
1.	1,8-Cineole		16.1	4.9	15.2	56.5	3.1	2.0
2.	*cis*-β-Ocimene	0.1			2.1			
3.	4-Methylene-1-(1-methylethyl)-3-Cyclo-hexene-1-ol			5.7				
4.	3-Carene				1.7			
5.	α-Cubebene			2.2	2.2	0.2		
6.	α-Elemene				1.3			
7.	α-Humelene			2.4				
8.	α-Terpinol	6.3				4		
9.	α-Phellandrene			8.1				
10.	α-Pinene	3.6	0.9	2.5	12.1	4.2	16	31.4
11.	β-Caryophyllene					1.2		
12.	β-Citronellal	3.2						
13.	β-Farnesol		11.1		2.8		10	
14.	β-Phellandrene	0.9					14.3	
15.	β-Pinene			2.6		0.2	1.5	2.5
16.	Amorphane						0.3	
17.	Benzaldehyde			0.8				
18.	Camphene				1.1	0.1	0.9	
19.	Camphor			1.3				
20.	Citrinyl acetate	2.8		9.1			2.8	13.2
21.	Citral	0.1						
22.	Citronellal	22.3						
23.	Citronellol	20						
24.	Citronellal oxime	1.4		1.0	3.6		1.4	
25.	Cymene-8-ol	0.3					1.6	
26.	Cyclopentanone							
27.	Eugenol	3.9	0.6	0.7	1.8		1.7	11.8
28.	Eucalyptol	0.1			3.3	0.2	1.1	8.1
29.	Digitoxigenine			1.8	1.7		0.4	
30.	Geranial	0.1			4.1			2.7
31.	Germacrene-D	7.5		2.5				
32.	Geranial oxime	1.9					4.2	
33.	Geranial	0.1	6.6	3.6		0.1	1.4	
34.	Geranial nitrile	0.7		1.1			3.6	
35.	Geranyl acetate				2.7			
36.	Globulol					2.4	0.6	
37.	Isopulegol	0.1			2.3		0.1	0.6
38.	Isosativene			3.7			4.5	
39.	Limonene	0.1		14.3	0.7	28	1.2	1.5
40.	Linalool	0.5	17	1.1	7.4	0.3	0.6	
41.	2-Methylprop-1-enyl-cyclohexa-1,5-diene			0.9				
42.	Myrtenal		0.6		8.1		9.2	
43.	Neriine				2.3	0.4	0.8	
44.	Neral			1.7	5.4		2.7	1.7
45.	Neral oxime				1,4			
46.	Paraldehyde		5.3		6.0		1.4	
47.	Paraldehyde nitrile	1.9		5.9	7.1		0.7	
48.	Patchoulene	9.4		3.0				
49.	*p*-Cymene		12.2				0.2	12.4
50.	Phenythyl acetate		7.1			0.2	1.8	1.3
51.	Pinocarveol							
52.	*p*-Ment-1-en-3,8-diol		5.1					
53.	Sabinene	4.2		1.4				
54.	Spathulenol			0.4				
55.	Solanone	0.3						
56.	Terpinene-4-ol	0.3	5.3	10.2			1.2	
57.	Verbenol						9.2	
58.	γ -Terpinene	0.7	10.7					
59.	γ-Terpinene	1			1.8		0.4	
60.	Trance-pinocarveol	1.1		6.8				
61.	γ-phellandrene	3.2						
62.	Ylangene	0.9						

### 2.4. Antimicrobial Activity

The EOs extracted from the seven *Eucalyptus* species showed great potential for their application as antimicrobial agents. The activities were determined by using three strains of bacteria and two strains of fungi.

#### 2.4.1. Antibacterial Activity

The EOs extracted from all seven *Eucalyptus* spp. showed antibacterial activity against *S. aureus, B. subtilis* and *E. coli* (Table 3). Maximum inhibition zones were observed for the *E. citriodora* EO against Gram positive bacteria (31 mm against *S. aureus* and 28 mm against *B. subtilis*). *E. melanophloia* and *E*. *citriodora* showed significant activity against the Gram negative bacterium *E. coli*.

**Table 3:** Comparative antimicrobial activities of essential oils of *Eucalyptus* species.

Microbial Species		Zones of Growth Inhibition ( in mm) by *Eucalyptus* Species								
		*E. citriodora*	*E. camaldulensis*	*E. crebra*	*E. tereticornis*	*E. globules*	*E. melanophloia*	*E. microtheca*	Amoxil	Nizoral
*Bacterial species*	*S. aureus*	31 ± 0.83 ^Aa^	21 ± 0.851 ^Ade^	23 ± 0.836 ^Acd^	22 ± 0.853 ^Ade^	28 ± 0.835 ^Acd^	26 ± 0.836 ^Abc^	16 ± 0.831 ^Ae^	22 ± 0.833 ^Aab^	-
	*B. subtilis*	28 ± 0.833 ^Aa^	24 ± 0.835 ^Ade^	21 ± 0.848 ^Acd^	18 ± 0.835 ^Ade^	17 ± 0.833 ^Acd^	22 ± 0.836 ^Abc^	20 ± 0.838 ^Ae^	28 ± 0.833 ^Aab^	-
	*E. coli*	15 ± 0.835 ^Ba^	10 ± 0.835 ^Bde^	12 ± 0.835 ^Bcd^	14 ± 0.836 ^Bde^	13 ± 0.83 ^Bcd^	16 ± 0.833 ^Bbc^	11 ± 0.835 ^Be^	20 ± 0.831 ^Bab^	-
*Fungal species*	*A. niger*	29 ± 0.831 ^Aa^	28 ± 0.835 ^Aab^	25 ± 0.836 ^Abc^	26 ± 0.833 ^Ab^	24 ± 0.835 ^Abc^	27 ± 0.835 ^Ac^	21 ± 0.835 ^Ac^	-	17 ± 0.835 ^Ac^
	*R. solani*	26 ± 0.836 ^Bb^	22 ± 0.829 ^Bab^	19 ± 0.835 ^Bbc^	21 ± 0.836 ^Bb^	20 ± 0.835 ^Bbc^	12 ± 0.835 ^Bc^	17 ± 0.838 ^Bc^	-	20 ± 0.835 ^Bc^

Each values is means of three. The capital letters represent significant difference in microbial species, while small letters represent significant difference in EOs.

However, all the species showed higher activity for the Gram positive bacteria as compared to the Gram negative bacteria. Gram positive bacteria were found to be more sensitive to *E. gillii* EO and extracts than Gram negative ones [22]. *E. oleosa* EO exhibited an interesting antibacterial activity against all microorganisms tested (*L. monocytogenes*, *S. aureus*, *E. coli*, *K. pneumoniae*, *S. cerevisiae*, *C. albicans*, *M. ramamnianus* and *A. ochraceus*). The activity in this study was better against Gram-positive bacteria except for *S. aureus* and *E. coli* [23]. Results have proved that *E. citriodora* EO is more potent in antibacterial activity than the other six species. The EOs of *Eucalyptus* have previously shown antimicrobial and antiplasmid activities [32]. Previous studies also report that EOs against food spoilage organisms and food-borne pathogens are slightly more active against Gram-positive than Gram-negative bacteria [33].

#### 2.4.2. Antifungal Activity

*E. citriodora* EO was found most effective against *A. niger* (29 mm) and *R. solani* (26 mm). Significant antimicrobial activity was shown by EO of *E. microtheca* against *A. niger* (21 mm) while EO of *E. melanophloia* was found least effective (12 mm) against *R. solani* (Table 3). The results showed that all the tested *Eucalyptus* EOs presented significant antifungal activity against *A. niger* and *R. solani* and had equal or more antifungal effect than amoxil and nizoral. These properties could be correlated to the chemical composition of the oils with good phenolic, alcoholic or aldehydic contents that may be correlated to the geographical distribution and environmental effect on production of phytochemicals in plants.

### 2.5. Antioxidant Activity

**Figure 1:**
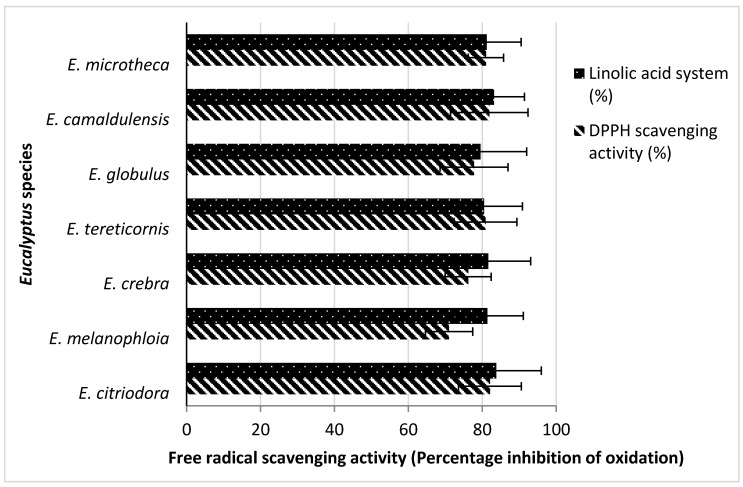
Antioxidant activities of *Eucalyptus* EOs through linoleic acid and diphenyl-1-picrylhydazyl radical scavenging assays. (Each value is mean of three).

The DPPH scavenging activity was highest in *E. citriodora* (82.1%), followed by *E. camaldulensis* (81.9%) and *E. microtheca* (81.8%) as compared to positive control BHT. The results are compared to the DPPH assay results for *E. oleosa* EO activity in the range of 12.0–52.8 mg/mL, whereas in the 2,2′-azinobis-3-ethylbenzothiazoline-6-sulfonate assay (176.5 ± 3.1 mg/L) it showed the best inhibition result [18]. All the EOs strongly suppressed the peroxide formation in linoleic acid system during incubation. The inhibition of oxidation of linoleic acid system was higher for the EOs of *E. citriodora* (83.8%) and *E. camaldulensis* (83.2%) than the other five *Eucalyptus* species (Figure 1). These findings are also supported by another study reporting 86.07% inhibition of linoleic acid for non-polar methanol extract of *E. sargentii* [34].

## 3. Experimental Section

### 3.1. Chemicals and Microbial Strains

All the chemicals used in this study were of analytical grade and purchased from Sigma Aldrich (Taufkirchen, Germany). *S. aureus*, *B. subtilis, E. coli, A. niger* and *R. solani* were obtained from and identified by the Department of Microbiology, University of Agriculture (Faisalabad, Pakistan). The bacterial strains were preserved and cultured separately in Lauria and Bertani (LB) media and fungal strains in Eggins and Pugh (E and P) medium broth by following the standard microbiological protocols. All microorganisms were stocked at −4 °C in standard conditions and were revived twice before use in the manipulations.

### 3.2. Collection of Samples

The fresh leaves from *E. citriodora, E. melanophloia*, *E. crebra*, *E. tereticornis*, *E. globulus*, *E. camaldulensis* and *E. microtheca* were collected from the Gutwala Forest Research Institute, (Faisalabad, Pakistan) in May 2014. All the leaves were washed with water to remove dirt particles and shade dried to reduce the moisture contents.

### 3.3. Procedure to Extract EOs

The EO was extracted from each plant material through a hydrodistillation method as described elsewhere [35]. In summary, the samples (leaves) were shade dried and a weighed amount (50 g) of crushed plant material was immediately charged to the distillation flask. Pressurized steam was circulated through the plant material. The vapors of the pure EO along with steam were condensed while passing through a water condenser and collected in a receiver flask kept in ice water in order to prevent the evaporation of the low boiling point constituents. The upper oily layer (2–3 mL) of condensed material was dissolved in diethyl ether (40 mL) and then separated from the distilled water component with the help of a separating funnel. Total EO was obtained after careful removal of the solvent by evaporation. The whole process of extraction was repeated till about 14–17 mL of oil was collected. The oils were dehydrated with Na_2_SO_4_ and the average percent yield was calculated. The EO was stored in a cool place away from heat and light.

### 3.4. Physiochemical Properties

The physiochemical properties of the oils like colour, odour, appearance, solubility in aqueous ethyl alcohol, boiling point, specific gravity at 20 °C and refractive index were determined by the methods described earlier [36,37,38,39].

### 3.5. Chemical Composition through GC-Mass Spectroscopy

Qualitative and quantitative determination of chemical composition for each EO was carried out through gas chromatography mass spectroscopy by using a GC-17A (Shimadzu, Kyoto, Japan) fitted with a DB-wax (30 mm × 0.25 mm) column (Agilent Technologies, Waldbornn, Germany) and a flame ionization detector (FID). Injector and detector temperatures were set at 250 °C and 260 °C, respectively. Column temperature was programmed from 90 °C for 2 min to 180 °C with a gradient of 2 °C/min. A second gradient was applied to 240 °C at 3 °C/min. Helium was used as a carrier gas at a flow rate of 30 mL/min at 150 psi. One µL sample of each EO was injected through the injector port. The chemical composition was determined by identifying the peaks with available data as standard and reported as a relative percentage of the total peak area. The quantitative measurements were made on chromatography station CSW 32 software of Data Apex (Prauge, Czech Republic, version 5.0).

### 3.6. Antimicrobial Activity

The antimicrobial activity of EOs was determined by using the disk agar diffusion method. The growing stock cultures were stabilized through various cycles for uniform growth. Sterilized Muller Hinton agar (Sigma-Aldrich, Taufkirchen, Germany) was cooled to 50 °C and inoculated with 100 µL fresh culture of each one of the above mentioned bacteria (10^5^–10^6^ bacteria/mL), separately. The inoculated medium (15 mL) was poured into sterilized petri dish of 9 cm diameter and swirled to distribute homogenously. Disks (9 mm diameter, Whatman filter paper no. 3) injected with 20 µL either oil or standard antibiotics (see below) were applied on solid agar medium. The plates were placed at 4 °C for 1–2 h and then incubated at 37 °C for 24 h. The zones of inhibition (including the size of the disk as well = 0.9 mm) on the media were measured with ruler [40]. Antifungal activity was determined on Sabouraud dextrose agar medium as described earlier [41]. The microorganisms were cultured in test tubes on the agar for 24 h. Fresh Sabouraud medium was inoculated with these spores to the desired concentration of cells (10^5^ spores/mL) and plates were prepared. Disks containing 20 µL EO were applied on the medium. The plates were incubated at 30 °C for three days and zones of inhibition were measured. The standard antibiotic control discs (containing 15 µg of the medicine on each disc) of Amoxil for bacteria and nizoral for fungi were used as controls, respectively.

The sensitivity of microorganism to each individual EO was determined by the diameter of the zones of inhibition with a small modification as described somewhere else [42]. Thus, the sensitivity was characterized as follows: not sensitive for total diameters smaller than 10 mm; sensitive for total diameters of 10–15 mm; very sensitive for total diameters of 16–20 mm; extremely sensitive for total diameters larger than 20 mm.

### 3.7. Antioxidant Activity of EOs

#### 3.7.1. DPPH Scavenging Activity

The free radical scavenging activities of the seven *Eucalyptus* EOs were assessed by measuring their scavenging abilities for stable 2,2′-diphenyl-1-picrylhydazyl DPPH radicals [43]. To do this, the samples (0.5 µg/mL) were mixed with 1 mL of 90 µM DPPH solution and made up with 95% methanol to a final volume of 4 mL. The mixture was incubated at 25 °C for 1 h and absorbance was measured at 515 nm using a spectrophotometer (U-2001, Hitachi, Tokyo, Japan). Butylated hydroxytoluene (BHT) was used as a positive control. All the samples were analyzed in triplicate. The free radical scavenging activity was determined as percent inhibition by using the following equation:

(1) $$\text{Inhibition }\left(\text{\%}\right)=100\;\left({\text{A}}_{\text{control}}-{\text{A}}_{\text{sample}}/{\text{A}}_{\text{control}}\right)$$

The antioxidant activities of EOs were expressed as IC_50_ values, which represented the concentrations of EOs that caused 50% neutralization of DPPH radicals and were calculated from the plot of inhibition percentage against concentration.

#### 3.7.2. Antioxidant Activity in Linoleic Acid System

The antioxidant activity of EOs were also determined using inhibition of linoleic acid oxidation [44]. To do this, the test samples (50 µL) were dissolved in ethanol (1 mL) then mixed with linoleic acid (52 µL), ethanol (4 mL) and 0.05 M sodium phosphate buffer (pH 7, 4 mL). The solution was incubated at 40 °C for 175 h. The colorimetric method was used to measure the extent of oxidation by peroxide value as described earlier [45]. For 0.2 mL sample solution, 10 mL of ethanol (75%), 0.2 mL of aqueous solution of ammonium thiocynate (30%) and 0.2 mL of ferrous chloride solution (20 mM in 3.5% HCl) were added sequentially. The contents were stirred for 3 min and the absorbance was measured at 500 nm. BHT was used as a positive control. The percentage of the inhibition of linoleic acid oxidation was calculated as follows:

(2) $$\begin{array}{cc} \text{\% inhibition of} & \text{linolic acid oxidation}\\ & =100-\left[\frac{\text{Abs}.\text{increase of sample at }175\text{ h}}{\text{Abs}.\text{increase of control at }175\text{ h}}\right]\times 100 \end{array}$$

### 3.8. Statistical Analysis

Statistical analysis was performed with the IBM Statistical Package of Social Sciences (SPSS, version 19, SPSS Inc., Chicago, IL, USA). Statistically significant differences were found by using two factorial ANOVA and significant results were represented at *p*-values < 0.001.

## 4. Conclusions

Applications of natural extracts are growing rapidly in the food, cosmetic and pharmaceutical industry. The present study systematically explored the potential of some local *Eucalyptus* species. The chemical composition of EOs from selected *Eucalyptus* species showed great variation. The chemical compounds of essentials oils reported in these species were also different from other reported *Eucalyptus* species. The antimicrobial potential of the EOs extracted from seven *Eucalyptus* species was higher against Gram positive bacteria than Gram negative ones and two types of fungi. All the results proved that these EOs were very effective and could be used in medicines, cosmetics, food and flavors industries. This report is so far first systematic study and comparison of EOs of local prevalent *Eucalyptus* species and their potential for uses in health and industries.
